# Supplementary material for: Comparative transcriptomic analysis reveals translationally relevant processes in mouse models of malaria
Source: eLife. 2022 Jan 10;11:e70763. doi: 10.7554/eLife.70763 (PMC8747512; doi:10.7554/eLife.70763)
Supplement: Supplementary file 1. — (A): The proportions of different leukocyte subtypes in the Lee et al. RNA-Seq data vs. the mouse models. (B): Details of the publicly available microarray data sets. (C): Comparison of host differential gene expression at onset of symptoms in a controlled human malaria infection study and at early stage illness in five mouse malaria models. (D): The Discordance-concordance analysis of the Boldt et al. Gabonese CH-UM vs Lee et al. Gambian children CM-UM differential expression analysis. (E): Gating strategy for defining WBC proportions in mouse blood. (F): Leucocyte proportions measured in whole blood by flow cytometry. (G): The genetic confirmation of the identity of the two different Plasmodium yoelii strains using the RNA-Seq reads in conjunction with the known single nucleotide variant. (H): The genetic confirmation of the two different Plasmodium berghei strains using the RNA-Seq reads in conjunction with four known single nucleotide variants. [file elife-70763-supp1.docx]

**
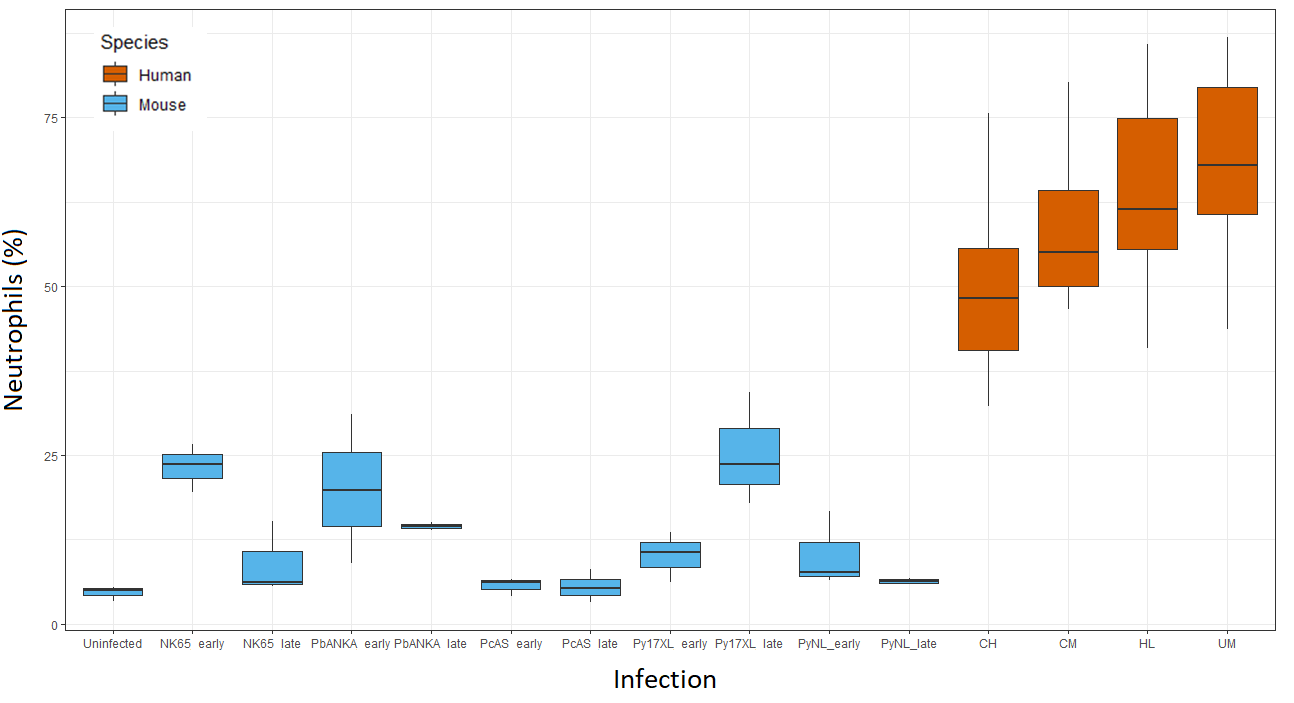

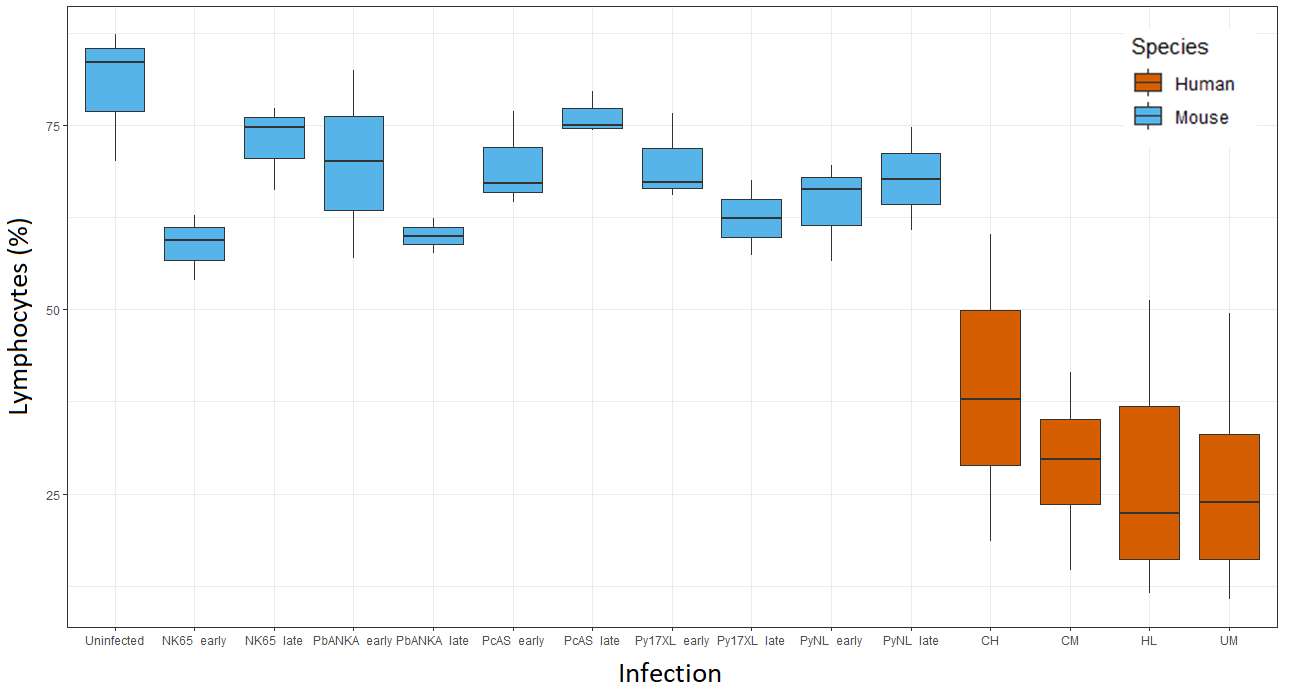
Supplementary File 1 Supplementary Figures and small tables**

**ii)**

**i)**

**
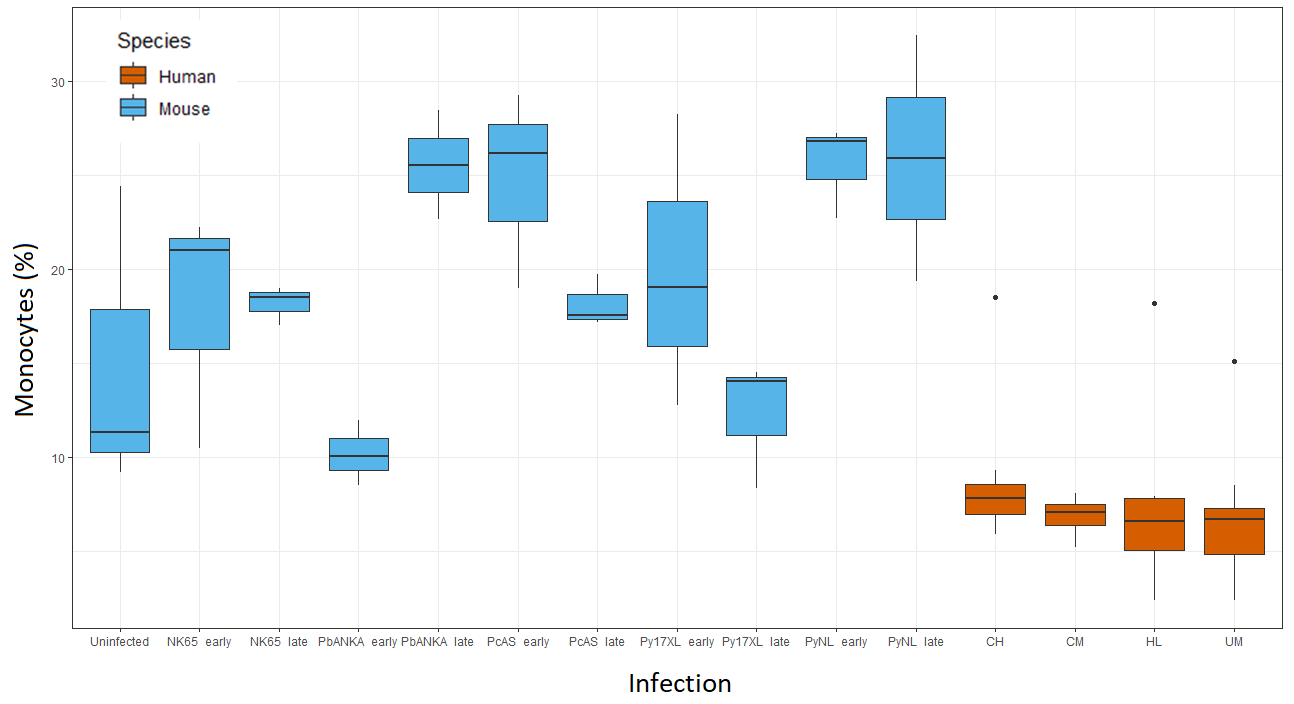
** **Supplementary File 1A: The proportions of different leukocyte subtypes in the Lee et al. RNA-Seq data vs. the mouse models**

**iii)**

The proportions of i) lymphocytes, ii) neutrophils and iii) monocytes measured by haematology analyser in the human RNA-Seq samples (Lee et al) were compared to the lymphocyte (sum of the B and CD4+T and CD8+T lymphocytes), neutrophils, and monocytes of the mouse RNA-Seq samples respectively. Due to missing values, for the lymphocyte comparison, 43 Lee et al. samples were used, 41 for neutrophils and 41 for monocytes. All 33 mouse RNA-Seq samples were used. These results were visualised as boxplots coloured by species. The mouse model abbreviations are as follows: PbNK65 (*P. berghei* *NK65*), PbANKA (*P. berghei* *ANKA*), PcAS (*P. chabaudi AS*), Py17XL (*P. yoelii* *17XL*) and Py17XNL (*P. yoelii* *17XNL*). Human phenotype abbreviations: cerebral malaria (CM), the combined phenotype of hyperlactatemia with cerebral malaria (CH), uncomplicated malaria (UM), hyperlactatemia (HL).

**Supplementary File 1B: Details of the publicly available microarray datasets**

| Accession Number | Citation | Clinical Phenotypes | Numbers of subjects | Gene Expression Platform | Background correction Method | Normalisation Method | Differential Expression Analysis Comparison(s) |
| --- | --- | --- | --- | --- | --- | --- | --- |
| GEO: GSE1124 | Boldt et al. 2019 | Healthy  Severe Anaemia (SA)  Cerebral Malaria (CM)  Uncomplicated Malaria (UM) | Healthy (n=5 pools of 4 patients), SA (n=5 pools of 4 patients), CM (n=5 pools of 4 patients), UM (n=5 pools of 4 patients) | Microarray  Affymetrix Human Genome U133A Array | normexp | Quantile Normalisation | CM-Healthy  SMA-Healthy  UM-Healthy |
| GEO: GSE34404 | Idaghdour et al. 2012 | Uncomplicated Malaria (UM)  Healthy | UM (n=93), Healthy (n=61)  1 symptomatic sample was removed because its age information was not available | Microarray  Illumina HumanHT-12 V4 Expression BeadChip | Imported as background corrected values. In the original paper background values were subtracted using the averaging of the negative control probes | Imported as normalised values, the original paper performed quantile normalisation | UM-Healthy |
| ArrayExpress: E-MTAB-6413 | Lee et al. 2018 | Uncomplicated Malaria (UM)  Cerebral Malaria (CM)  Hyperlactatemia (HL)  Cerebral Malaria and Hyperlactatemia (CH) | UM (n=50)  CM (n=14)  HL (n=16)  CH (n=26) | RNA-Seq  Illumina HiSeq 2500 | Not applicable | Imported the results of the differential expression analysis. The expression values were normalised by the originally paper using the trimmed mean of M-values method | HL-UM  CM-UM  CH–UM |
| GEO: GSE132050 | Milne et al., 2021 | day -1  day of diagnosis | N=28, 2 samples per individual (day -1 and day of diagnosis)  One individual from the publicly available dataset (Malaysia sample) was removed. | Affymetrix Human Transcriptome Array 2.0 | rma() function  getMainprobes()  annotateEset() | rma() function to perform quantile normalisation | Day of diagnosis-day -1 |
| GEO: GSE93631 | Talavera-López et al., 2019 | naive samples (Day 12)  PcAS (*P. chabaudi AS*) Day 4 (early stage of infection)  PcAS day 8 (late stage of infection) | n=3 per phenotype | Illumina MouseWG-6 v2.0 expression beadchip | Not applicable | Imported as partially processed data. An additional quantile normalisation step was performed | PcAS day 8 (late)-PcAS day 4 (early) |

**Supplementary File 1C: Comparison of host differential gene expression at onset of symptoms in a controlled human malaria infection study and at early-stage illness in five mouse malaria models.**

i) Principal Component Analysis (PCA) generated using rank-normalised log-fold change values from the human and mouse differential expression analyses. Only genes with 1:1 mouse and human orthologs and with absolute logFC value greater than 1 in the corresponding human comparison were included. Comparison of changes in gene expression in the mouse models (uninfected vs early in infection) with the day of first symptoms (uncomplicated mild symptoms, noted as “day of diagnosis” in the original paper) vs the day before infection for the same individuals (PfUM) in malaria naïve individuals. The percentage of the total variation explained by principal components 1 and 2 are shown in the axis labels. Greyscale heatmaps parallel to each axis show the contributions of the 10 genes contributing most to the corresponding PC. ii) Heatmaps that show the logFC values for the 20 genes with the greatest absolute log fold change values in the human differential gene expression analysis, and their orthologs in each mouse model, corresponding to the analyses. Mouse models are ordered left to right in order of increasing dissimilarity to the human disease, based on the Euclidian distance calculated from all principal components (Supplementary File 13). The rows (genes) are ordered by absolute log-fold change in the human comparison in descending order. Mouse models: n=3 for early and n=3 late for each mouse model. The mouse model abbreviations are as follows: PbNK65 (*P. berghei NK65*), PbANKA (*P. berghei ANKA*), PcAS (*P. chabaudi AS*), Py17XL (*P. yoelii 17XL*) and Py17XNL (*P. yoelii 17XNL*). Human subjects, n=14, each with paired samples before infection and at onset of symptoms.


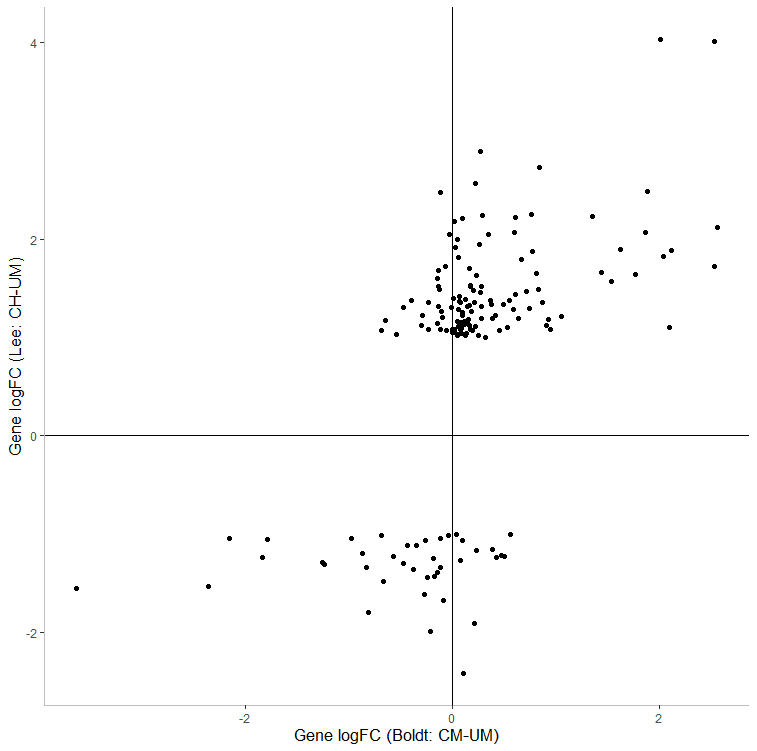


**Supplementary File 1D: The Discordance-concordance analysis of the Boldt et al. Gabonese CH-UM vs Lee et al. Gambian children CM-UM differential expression analysis.**

Genes with an absolute log-fold change value greater than 1 in the Lee CM-UM comparison were used (161 genes) in this analysis. This illustrates a concordance of gene expression change of 77.6% and discordance of 22.4% between analyses. Lee et al. CH n=12, UM n=21. Boldt et al. n=5 pools of samples (each pool contained RNA from 4 Gabonese children with the same phenotype). Abbreviations: cerebral malaria (CM), the combined phenotype of hyperlactatemia with cerebral malaria (CH), uncomplicated malaria (UM).


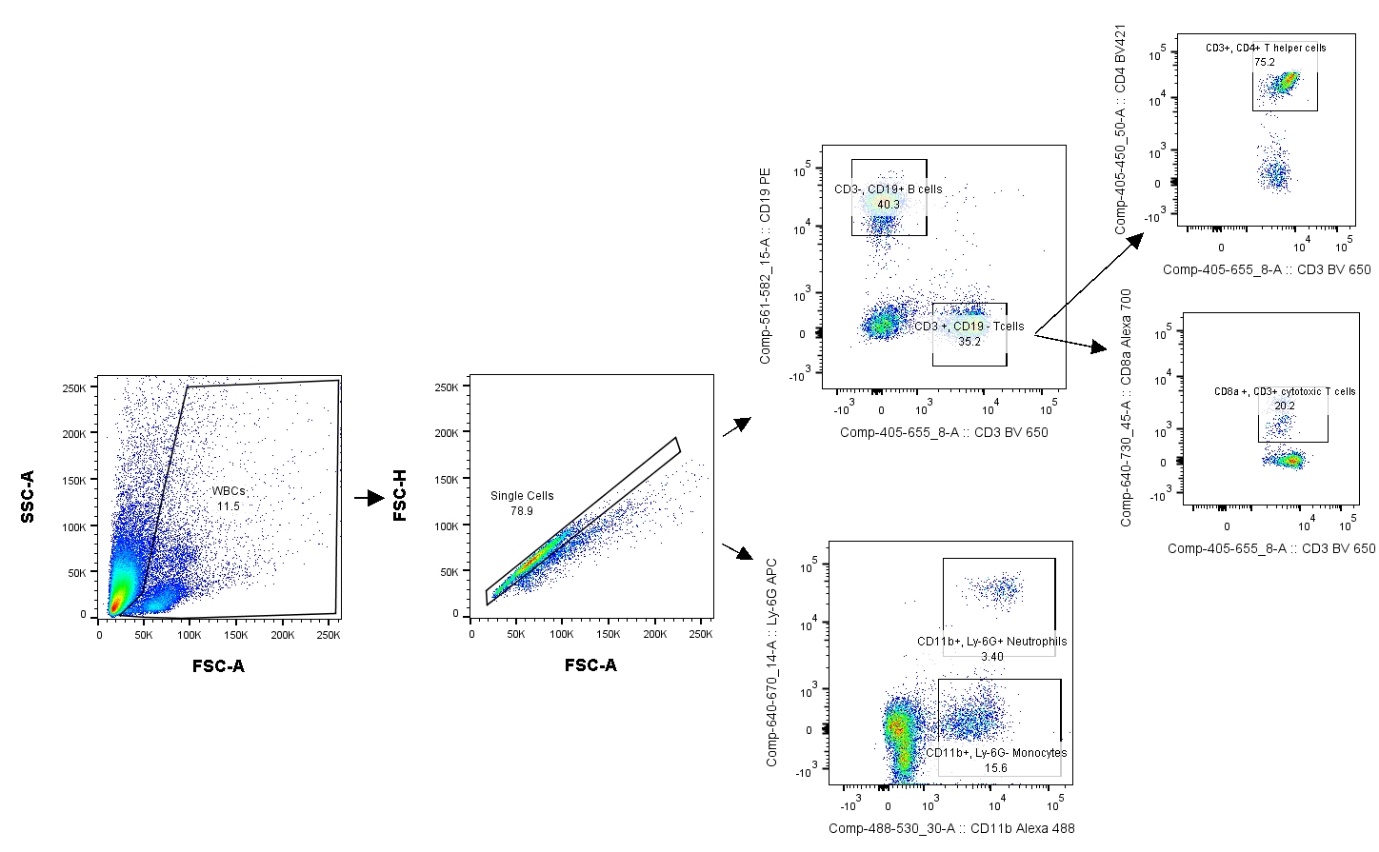


**Supplementary File 1E: Gating strategy for defining WBC proportions in mouse blood**

The strategy included gating around the WBC population excluding red blood cells that did not lyse and debris using FSC-A/SSC-A. Then doublets were excluded with FSC-A and FSC-H. Using different combinations of antibodies or antibody/ SSC-A proportions of the populations of interest were defined. T cells gating: CD3 +, CD19-; T helper cells: CD8a- , CD4+; cytotoxic T cells: CD8a+, CD4-; B cells: CD3-, CD19+; Monocytes: CD11b +, Ly-6G-; Neutrophils: Ly-6G+, CD11b +.


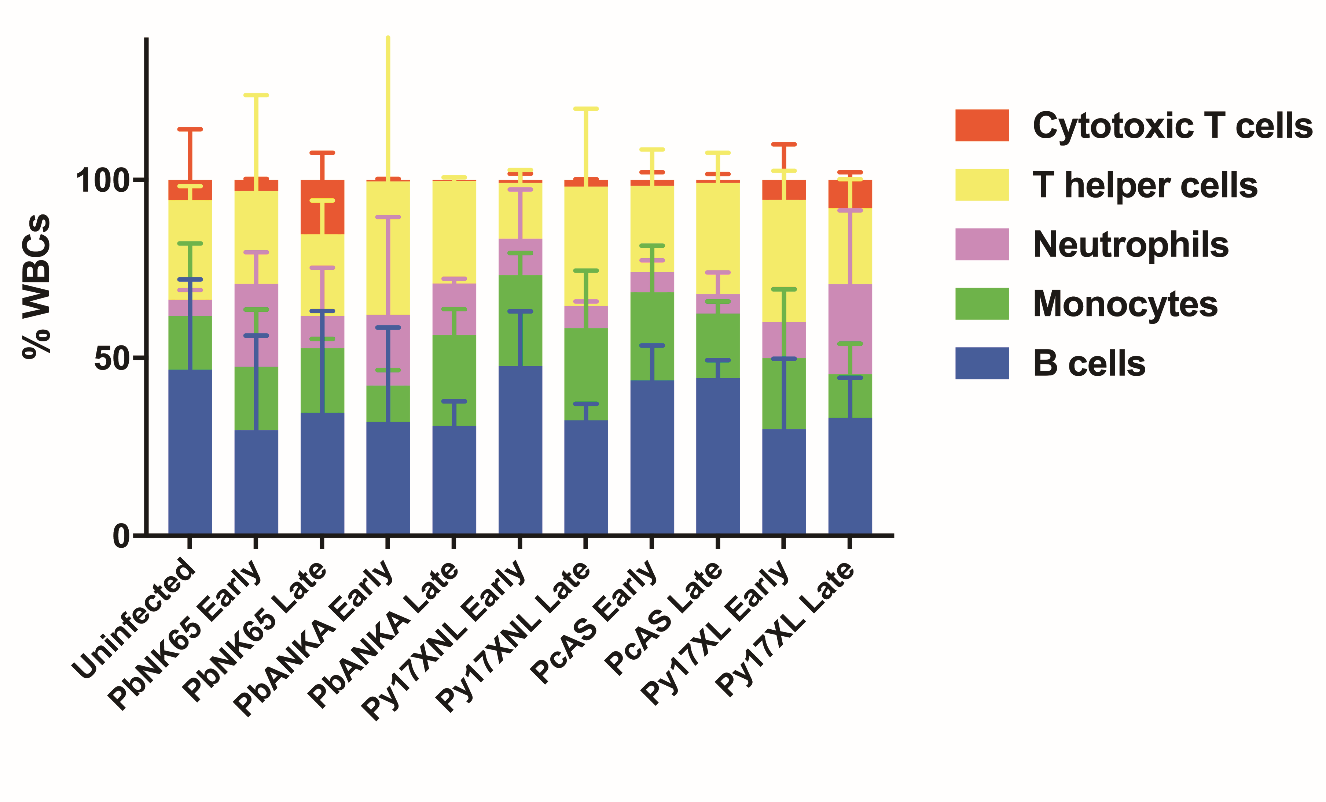


**Supplementary File 1F: Leucocyte proportions measured in whole blood by flow cytometry.**

8-week-old female wild type C57BL/6 mice infected with: *P. yoelii 17XL*, *P. berghei ANKA*, *P. berghei NK65*, *P. yoelii 17XNL*, *P. chabaudi AS*, and uninfected controls are presented here. Proportions of B cells, monocytes, neutrophils, T helper cells and cytotoxic T cells were measured at the early and late time point of each infection and compared to uninfected mice. n=3 for early and n=3 for late time point in each mouse model; n=3 for uninfected mice. Bars show mean with 95% CI. The mouse model abbreviations are as follows: PbNK65 (*P. berghei NK65*), PbANKA (*P. berghei ANKA*), PcAS (*P. chabaudi AS*), Py17XL (*P. yoelii 17XL*) and Py17XNL (*P. yoelii 17XNL*).

**
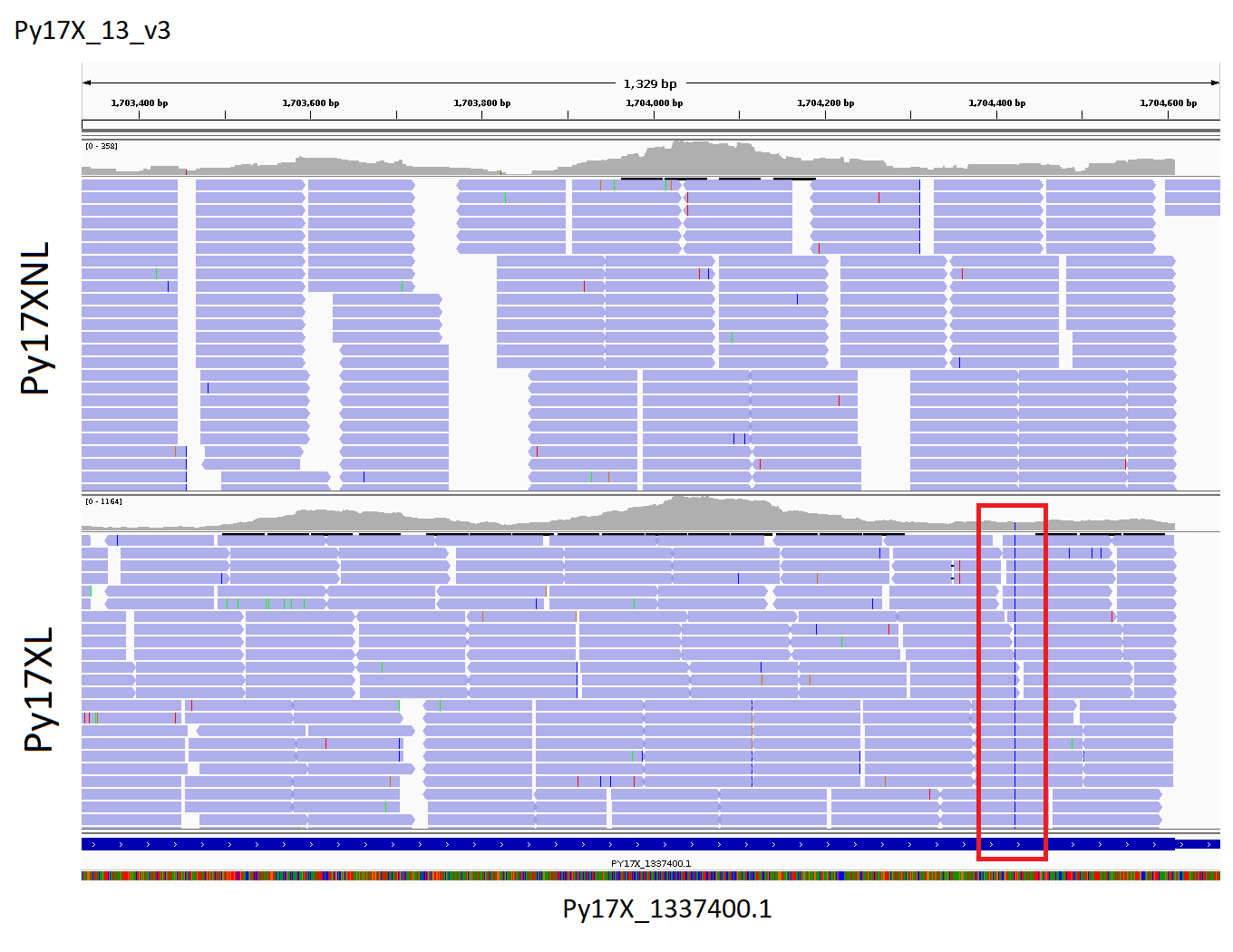
 Supplementary File 1G: The genetic confirmation of the identity of the two different *Plasmodium yoelii* strains using the RNA-Seq reads in conjuncture with the known single nucleotide variant.**

A single genetic variant in the PyEBL gene (PY17X_1337400) gene resulting from a SNP is thought to be responsible for the phenotypic differences between the lethal (Py17XL) and non-lethal (Py17XNL) *P. yoelii* strains (Otsuki et al., 2009). To confirm the presence of this variant in the lethal strain and absence in the non-lethal the chromosomal location of the SNP was identified as 1,704,423 and IGV (Robinson et al., 2011) was used to examine this region using the Py17XNL genome and annotation as a reference. This IGV output shows the presence of this mismatch variant in an example Py17XL late-stage sample and absence in an example late sample with the PyNL strain. This is highlighted in the red box on the IGV output shown. Abbreviations: Py17XL (*P. yoelii* *17XL*) and Py17XNL (*P. yoelii* *17XNL*).


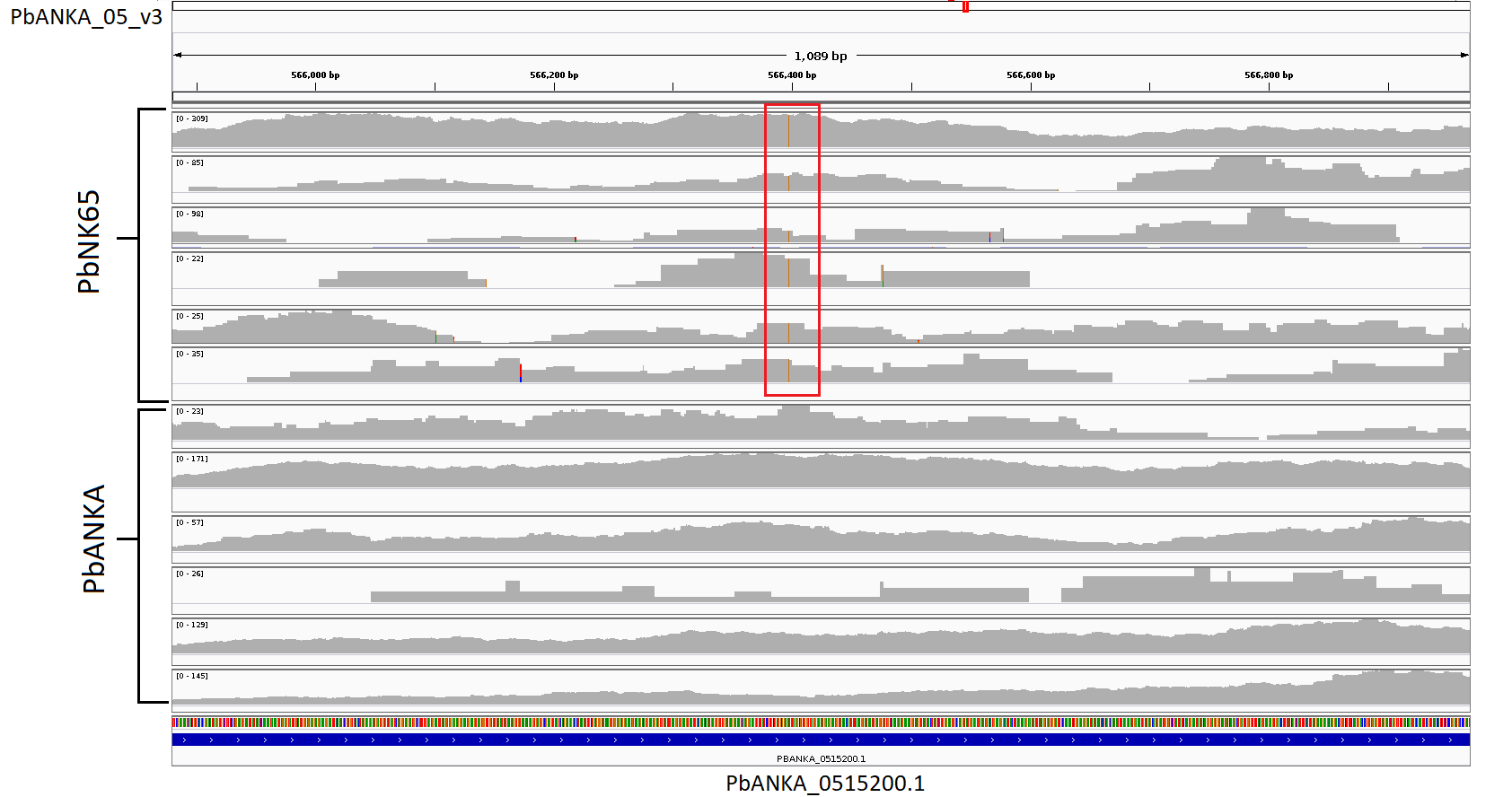

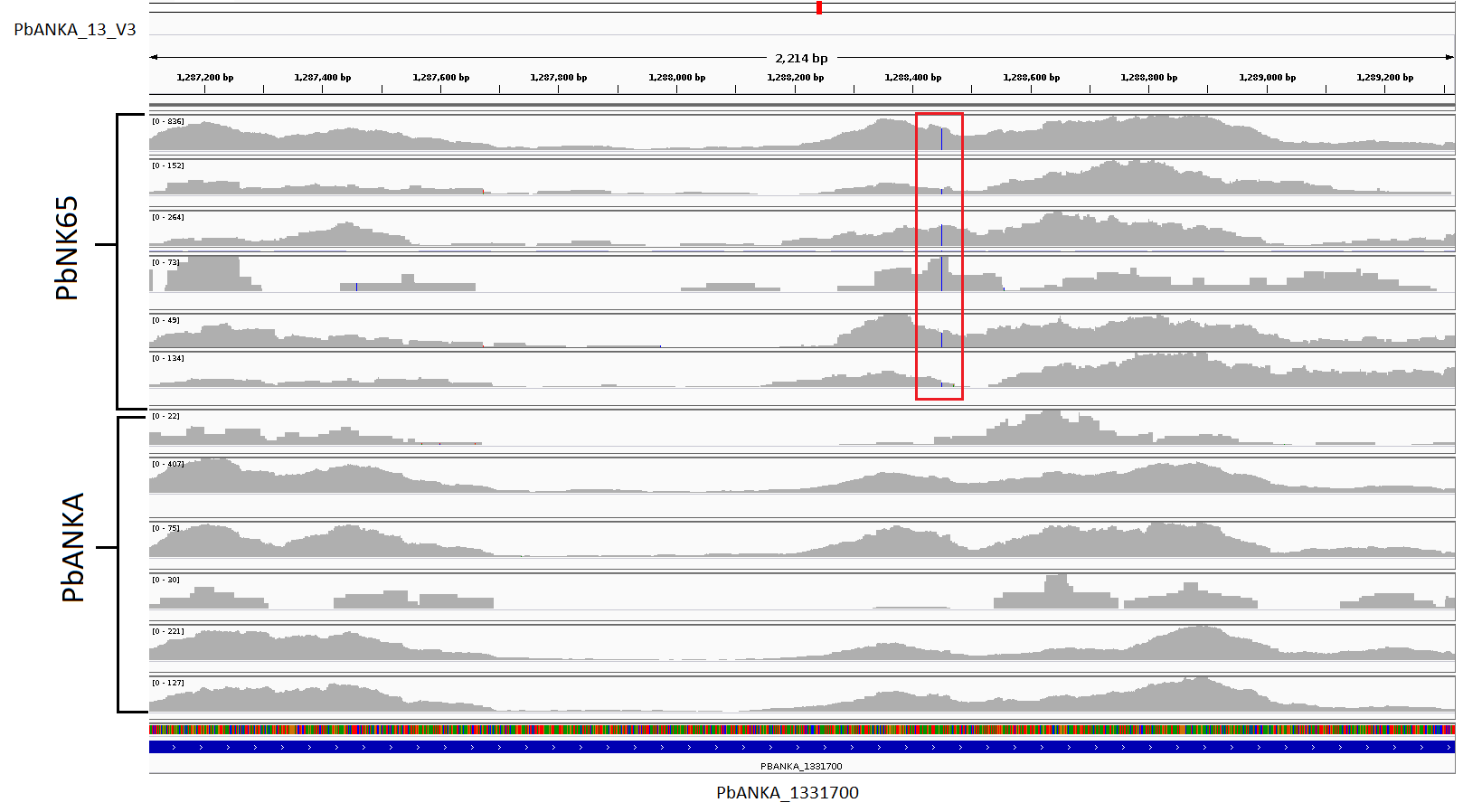


**ii)**

**i)**


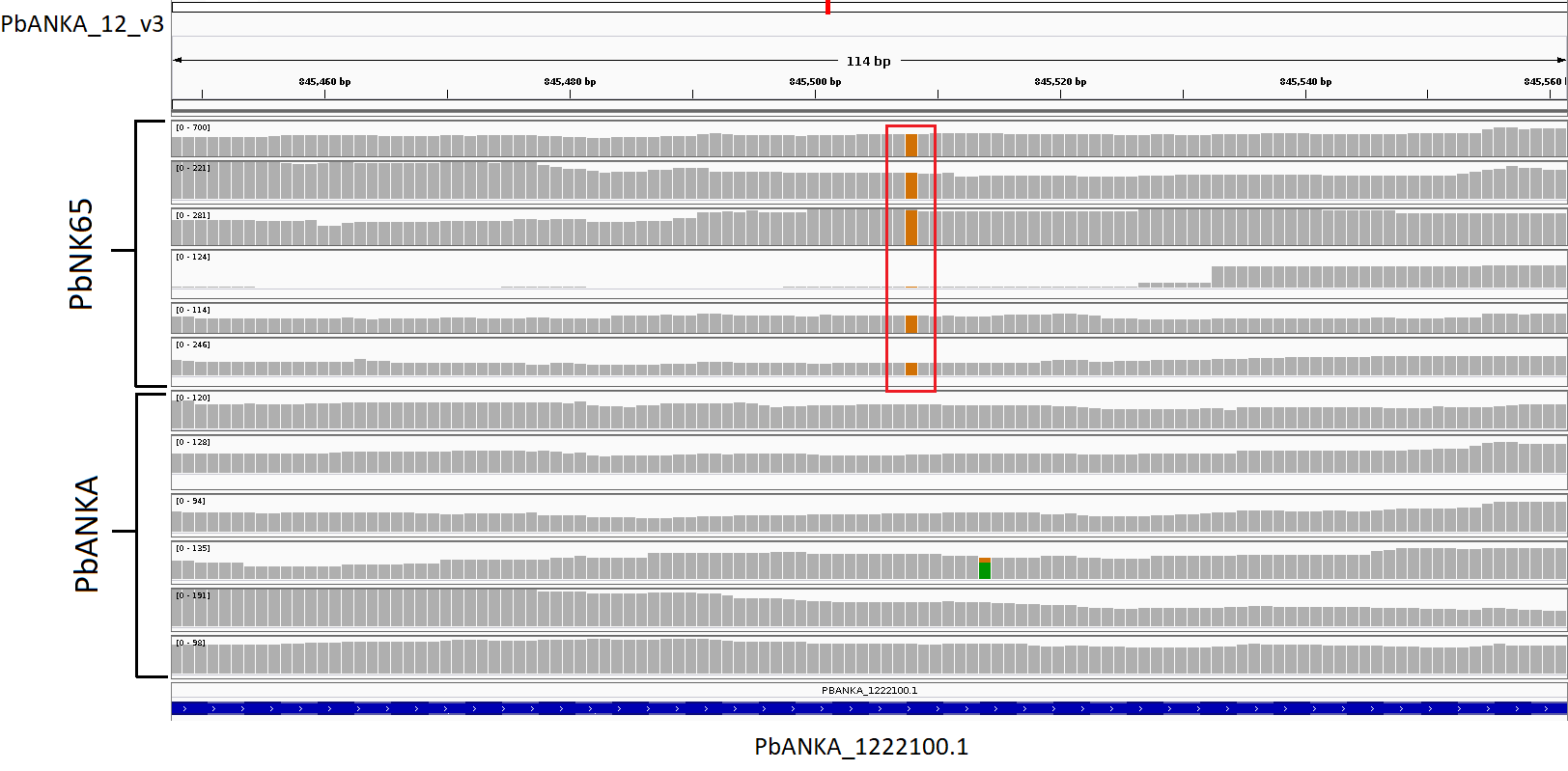

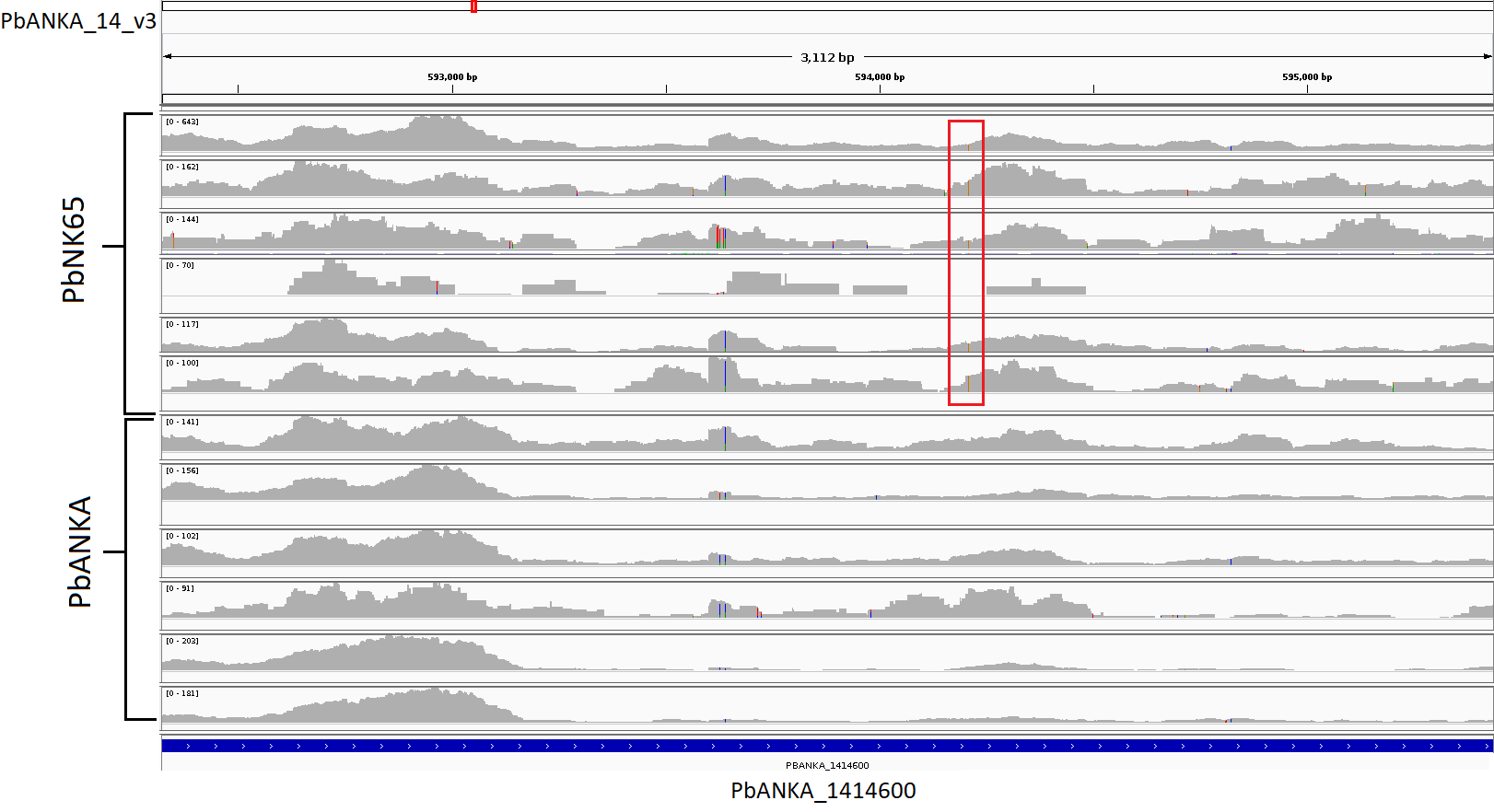


**iv)**

**iii)**

**Supplementary File 1H: The genetic conformation of the two different *Plasmodium* *berghei* strains using the RNA-Seq reads in conjunction with 4 known single nucleotide variants**

The variants in the i) PbANKA_1331700, ii) PbANKA_0515200.1, iii) PbANKA_1414600, and iv) PbANKA_1222100.1 genes are SNPs that are thought to distinguish the PbANKA from the PbNK65 strains (Akkaya et al., 2020). These have been examined in IGV (Robinson et al., 2011) for all 6 samples per strain relative to the PbANKA genome and annotation. Thus, the presence as mismatches (highlighted in the red box) in the NK65 and absence in PbANKA is illustrated. Abbreviations: PbNK65 (*P. berghei NK65*), PbANKA (*P. berghei ANKA*)
