# Supplementary material for: Comparative transcriptomic analysis reveals translationally relevant processes in mouse models of malaria
Source: eLife. 2022 Jan 10;11:e70763. doi: 10.7554/eLife.70763 (PMC8747512; doi:10.7554/eLife.70763)
Supplement: Transparent reporting form [file elife-70763-transrepform1.docx]

***eLife’s* transparent reporting form**

We encourage authors to provide detailed information *within their submission* to facilitate the interpretation and replication of experiments. Authors can upload supporting documentation to indicate the use of appropriate reporting guidelines for health-related research (see [EQUATOR Network](http://www.equator-network.org/%20)), life science research (see the [BioSharing Information Resource](https://biosharing.org/)), or the [ARRIVE guidelines](http://www.plosbiology.org/article/info:doi/10.1371/journal.pbio.1000412) for reporting work involving animal research. Where applicable, authors should refer to any relevant reporting standards documents in this form.

If you have any questions, please consult our Journal Policies and/or contact us: [editorial@elifesciences.org](mailto:editorial@elifesciences.org).

**Sample-size estimation**

- You should state whether an appropriate sample size was computed when the study was being designed
- You should state the statistical method of sample size computation and any required assumptions
- If no explicit power analysis was used, you should describe how you decided what sample (replicate) size (number) to use

Please outline where this information can be found within the submission (e.g., sections or figure legends), or explain why this information doesn’t apply to your submission:

Methods: Experimental design / Animals and procedures / Analysis of Human Microarray Datasets / RNA isolation from mouse blood

**Replicates**

- You should report how often each experiment was performed
- You should include a definition of biological versus technical replication
- The data obtained should be provided and sufficient information should be provided to indicate the number of independent biological and/or technical replicates
- If you encountered any outliers, you should describe how these were handled
- Criteria for exclusion/inclusion of data should be clearly stated
- High-throughput sequence data should be uploaded before submission, with a private link for reviewers provided (these are available from both GEO and ArrayExpress)

Please outline where this information can be found within the submission (e.g., sections or figure legends), or explain why this information doesn’t apply to your submission:

Methods: Experimental design / Animals and procedures / Analysis of Human Microarray Datasets / RNA isolation from mouse blood, Results: Figure legends

**Statistical reporting**

- Statistical analysis methods should be described and justified
- Raw data should be presented in figures whenever informative to do so (typically when N per group is less than 10)
- For each experiment, you should identify the statistical tests used, exact values of N, definitions of center, methods of multiple test correction, and dispersion and precision measures (e.g., mean, median, SD, SEM, confidence intervals; and, for the major substantive results, a measure of effect size (e.g., Pearson's r, Cohen's d)
- Report exact p-values wherever possible alongside the summary statistics and 95% confidence intervals. These should be reported for all key questions and not only when the p-value is less than 0.05.

Please outline where this information can be found within the submission (e.g., sections or figure legends), or explain why this information doesn’t apply to your submission:

Methods: Statistical tests / Analysis of Human Microarray Datasets / Mouse Differential gene expression analysis, Results: Figure legends

(For large datasets, or papers with a very large number of statistical tests, you may upload a single table file with tests, Ns, etc., with reference to sections in the manuscript.)

**Group allocation**

- Indicate how samples were allocated into experimental groups (in the case of clinical studies, please specify allocation to treatment method); if randomization was used, please also state if restricted randomization was applied
- Indicate if masking was used during group allocation, data collection and/or data analysis

Please outline where this information can be found within the submission (e.g., sections or figure legends), or explain why this information doesn’t apply to your submission:

Methods: Experimental design / Animals and procedures

**Additional data files (“source data”)**

- We encourage you to upload relevant additional data files, such as numerical data that are represented as a graph in a figure, or as a summary table
- Where provided, these should be in the most useful format, and they can be uploaded as “Source data” files linked to a main figure or table
- Include model definition files including the full list of parameters used
- Include code used for data analysis (e.g., R, MatLab)
- Avoid stating that data files are “available upon request”

Please indicate the figures or tables for which source data files have been provided:

**Figure 1**: Course of infection in five mouse malaria models

Figure 1- Figure Supplement 1 Severity scoring

Figure 1 - Source Data 1: Individual mouse parasitemia and weights

Supplementary files:

Supplementary File 17_severity scoring

**Figure 2:** Comparison of host differential gene expression in human uncomplicated malaria and early-stage illness in five mouse malaria models.

Figure 2- Figure Supplement 1, 2, 3, 4 Additional Heatmaps for figure 2

Supplementary files:

Input:

Supplementary File 6 Idaghdour et al 2012 Differential Expression Analysis

Supplementary File 7 Boldt et al 2019 Differential Expression Analysis

Supplementary File 8 Human Mouse Orthologs

Output:

Supplementary File 13 Euclidean Distances

Supplementary File 23 PCA input standardised logFC values

Supplementary File 24 Genes contributing to PC1 and PC2

**Figure 3:** Comparison of host differential gene expression in three severe malaria phenotypes in Gambian Children and five mouse malaria models.

Figure 3- Figure Supplement 1, 2, 3, 4, 5, 6 Additional Heatmaps for figure 3

Supplementary files:

Input:

Supplementary File 8 Human Mouse Orthologs

Supplementary File 12 Mouse Differential Expression Analysis

Supplementary File 14 Lee et al 2018 Differential Expression Analysis

Output:

Supplementary File 13 Euclidean Distances

Supplementary File 23 PCA input standardised logFC values

Supplementary File 24 Genes contributing to PC1 and PC2

**Figure 4:** Comparison of host differential gene expression in two severe malaria phenotypes in Gabonese Children and five mouse malaria models.

Figure 4- Figure Supplement 1, 2, 3, 4 Additional Heatmaps for figure 4

Supplementary files:

Input:

Supplementary File 8 Human Mouse Orthologs

Supplementary File 12 Mouse Differential Expression Analysis

Supplementary File 7 Boldt et al 2019 Differential Expression Analysis

Output:

Supplementary File 13 Euclidean Distances

Supplementary File 23 PCA input standardised logFC values

Supplementary File 24 Genes contributing to PC1 and PC2

**Figure 5:** Pathophysiological features of rodent malaria infections.

Source Data:

Figure 5 - Source Data 1: Individual mouse lactate measurements & erythrocyte counts
